# Interphase fluorescence in situ hybridization analysis of CD19‐selected cells: Utility in detecting disease in post‐therapy samples of B‐cell neoplasms

**DOI:** 10.1002/cam4.3853

**Published:** 2021-03-15

**Authors:** Andrew M. Parrott, Vundavalli V. Murty, Caitlin Walsh, Alecia Christiano, Govind Bhagat, Bachir Alobeid

**Affiliations:** ^1^ Department of Pathology and Cell Biology Columbia University Irving Medical Center and NewYork‐Presbyterian Hospital New York NY USA

**Keywords:** B‐cell, CD19‐selection, cytogenetics, FISH, flow cytometry, karyotype, leukemia, lymphoma, measurable residual disease, minimal disease, neoplasm, post‐therapy

## Abstract

**Context:**

The detection of low‐level persistent or relapsed B‐cell neoplasms, particularly post‐therapy, can be challenging, often requiring multiple testing modalities.

**Objective:**

Here we investigate the utility of CD19‐based selection of neoplastic B‐cells (CD19S) as an enrichment strategy to improve the detection rate of cytogenetic abnormalities in post‐therapy samples of B‐cell neoplasms, especially those with low‐level disease.

**Design:**

In a cohort largely comprised of post‐therapy B‐ALL and CLL samples, we performed fluorescence in situ hybridization (FISH) analysis on CD19‐selected cells (CD19S FISH) in 128 specimens from 88 patients, and on non‐selected cells (NS FISH) in a subset of cases. The FISH findings were compared with the concurrent flow cytometry (FC) results in all samples and molecular analysis in a subset.

**Results:**

CD19S FISH was able to detect cytogenetic aberrations in 86.0% of post‐therapy samples with evidence of disease as determined by routine or MRD FC, compared to 59.1% of samples by NS FISH. CD19S FISH detected significantly higher percentages of positive cells compared to NS FISH (*p* < 0.001). Importantly, CD19S FISH enabled the detection of emergent subclones (clonal evolution) associated with poor prognosis.

**Conclusions:**

CD19S FISH can be useful in daily diagnostic practice. Compared to NS FISH, CD19S FISH is quantitatively and qualitatively superior for the detection of cytogenetic aberrations in B‐cell neoplasms, which are important for risk stratification and optimal management of patients with B‐cell neoplasms, especially in the relapsed setting. Although CD19S FISH has a diagnostic sensitivity inferior to that of MRD FC, the sensitivity of this modality is comparable to routine FC for the evaluation of low‐level disease in the post‐therapy setting. Moreover, CD19S samples are invaluable for additional molecular and genetic analyses.

## Graphical abstract

A systematic comparison of CD19‐selected (CD19S) FISH versus non‐selected FISH analysis establishes superiority (qualitative and quantitative) of the former modality in detecting post‐therapy persistence or relapse of B‐cell neoplasms. CD19S FISH can complement flow cytometric evaluation for the detection of low‐level involvement in B‐cell neoplasms and particularly in the detection of post‐therapy recurrent/emerging small subclones. Flow cytometry detection of <1% positive cells was predictive of a “negative” non‐selected FISH outcome; further suggesting the benefit of FISH analysis on CD19S cells post‐therapy.

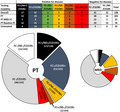

**Key points**

1. A systematic comparison of CD19S FISH versus NS FISH analysis establishes superiority (qualitative and quantitative) of the former modality in detecting post‐therapy persistence or relapse of B‐cell neoplasms.
2. CD19S FISH can complement flow cytometric evaluation for the detection of low‐level involvement in B‐cell neoplasms and particularly in the detection of post‐therapy recurrent/emerging small subclones.
3. Flow cytometry detection of <1% positive cells was predictive of a “negative” non‐selected FISH outcome; further suggesting the benefit of FISH analysis on CD19‐selected cells post‐therapy.

## 1 INTRODUCTION

Detection of cytogenetic or molecular aberrations plays an integral role in the diagnosis, classification, and management of B‐cell neoplasms.^1^ Recurrent genetic aberrations are also important for risk stratification of patients with B‐cell neoplasms, particularly in chronic lymphocytic leukemia/small lymphocytic lymphoma (CLL/SLL)^2^ and B lymphoblastic leukemia/lymphoma (B‐ALL).^3^ However, the ability to detect such aberrations in peripheral blood, bone marrow, and other samples with low‐level disease can be challenging, particularly post‐therapy, to assess for measurable residual disease (MRD) or early relapse. Currently, flow cytometry (FC) is the standard method for detecting MRD in B‐ALL, plasma cell neoplasms, and acute myeloid leukemia. Another current method used in the detection of B‐ALL MRD is polymerase chain reaction (PCR) analysis.^4^

A number of methods have been devised to separate and enrich neoplastic cells from complex cellular mixtures that target cell and cancer‐specific surface antigens.^5^ Of these, magnetic‐activated cell sorting (MACS) has become the standard modality due to its affordability, ease of use, high‐throughput, and high purity for isolating different cell types through the use of single or multiple antibody‐coated magnetic beads.^6^, ^7^ Sorted cells can then be interrogated by cytogenetic or molecular techniques, such as interphase fluorescence in situ hybridization (FISH) and next‐generation sequencing. Utilization of CD138‐positive MACS coupled with interphase FISH is currently widely used for the detection of recurrent cytogenetic aberrations in plasma cell neoplasms.^8^, ^9^ A few prior studies have documented the use of CD19‐positive MACS to evaluate B‐cell neoplasms, mostly CLL/SLL, in post‐therapy settings,^10^, ^11^ for determining prognostic markers,^12^ and characterizing the mutational landscapes of B‐cell neoplasms.^13^, ^14^ Sporadic reports have described the clinical application of CD19 selection coupled with interphase FISH analysis for the evaluation of CLL/SLL.^15^, ^16^ However, to the best of our knowledge, no previously published studies have systematically and comprehensively evaluated the utility of FISH analysis on CD19‐selected cells in the detection of B‐cell neoplasms in routine diagnostic practice.

The aim of the present study was to compare the findings of interphase FISH analysis performed on CD19‐selected cells (CD19S FISH) with those of FISH performed on non‐selected cells (NS FISH), and also compare the results with concurrent FC analysis (routine and MRD FC) and molecular polymerase chain reaction (PCR) analysis in various types of mature and immature CD19‐positive B‐cell neoplasms. We show that CD19S FISH is quantitatively and qualitatively superior to NS FISH for detecting cytogenetic aberrations as well as in identifying emerging subclones, especially post‐therapy. Furthermore, the sensitivity of CD19S FISH is comparable to routine FC for monitoring low‐level disease persistence or relapse.

## 2 MATERIALS AND METHODS

### 2.1 Case selection

We retrospectively analyzed consecutive pediatric and adult bone marrow aspirate and peripheral blood samples involved by B‐cell neoplasms over a period of 23 months (June 2018‐April 2020). The criteria for case selection included: i) mature and immature B‐cell neoplasms, with either a previously established diagnosis at our institution or newly determined from diagnostic/untreated samples, ii) known chromosomal aberrations detectable by FISH, and iii) concomitant evaluation of samples by either routine or MRD FC. Pathological diagnoses and results of all routine morphology and ancillary studies were retrieved from our departmental database. Disease detection by FC analysis was considered the “gold‐standard” for the purpose of this study. The study was performed in accordance with the principles of the Declaration of Helsinki and a protocol approved by the Institutional Review Board of Columbia University Human Research Protection Office.

### 2.2 Flow cytometry

Routine multi‐color FC or B‐ALL MRD FC analysis, conducted on a BD FACSCanto II cell analyzer (Becton, Dickinson and Company, Franklin Lakes, NJ), was performed on bone marrow aspirate and peripheral blood samples to evaluate B‐cell neoplasms. All samples were processed as previously described.^17^ Four‐ and eight‐color FC antibody panels were used, including B‐cell (CD19, CD20, CD45, surface κ, surface λ, CD10, CD5, CD43, CD103, CD23, CD38, FMC7, CD11c, CD30, CD34, CD52, IgM, IgD, cytoplasmic IgM, cytoplasmic CD79a, and TdT), and plasma cell (CD138, CD38, CD117, CD56, CD27, CD28, CD45, CD19, CD20, cytoplasmic κ, and cytoplasmic λ) panels. The B‐ALL MRD panel comprised CD20, CD10, CD38, CD19, CD58, CD45, CD9, CD13&33, CD34, CD3 and CD71 markers. All antibodies were obtained from BD Biosciences (Franklin Lakes, NJ). Data collection and analysis were performed using BD FACSDiva software (BD Bioscience, San Jose, CA) and FCS Express version 5 (DeNovo Software, Pasadena, CA), respectively. The accepted limit of detection for routine FC in our laboratory was 0.1% and for B‐ALL MRD FC was 1/10,000 events. The average number of cells per tube analyzed by routine FC in bone marrow specimens in this study was approximately 41,000, and in peripheral blood specimens was approximately 16,000, while the average number of events collected by MRD was approximately 1,400,000.

### 2.3 Magnetic‐bead based Cell selection

CD19 magnetic‐bead‐selection and enrichment of cells of interest was performed with the RoboSep‐S instrument (STEMCELL Technologies, Vancouver, Canada) according to the manufacturer's protocol. Briefly, the sample was diluted 5‐fold using RoboSep dilution buffer (STEMCELL Technologies, Vancouver, Canada), mixed gently, and spun at 300 g for 10 minutes, followed by the aspiration of the supernatant. The sample was then diluted to the original volume with RoboSep buffer and mixed well. If required, the sample was filtered through a pre‐wetted 70 µm strainer (Fisher Scientific, Waltham, MA) to remove bone fragments and cell aggregates or debris. The sample was diluted 1:1 in EasySep RBC buffer (STEMCELL Technologies, Vancouver, Canada), transferred to a 14 ml centrifuge tube, and processed on the RoboSep‐S instrument using the RoboSep HLA Chim WB CD19 Positive Selection Kit (STEMCELL Technologies, Vancouver, Canada). In‐house magnetic‐bead selection achieved CD19+ cell purities within the stated range of the manufacturer (94.3%–99.6%).

### 2.4 Cytogenetic/FISH analysis

FISH analysis was performed on CD19S and NS cells using standard protocols. Briefly, CD19S and NS cells were subjected to hypotonic treatment for 10 minutes, followed by fixing the cells with fresh 3:1 methanol‐acetic acid fixative three times, and preparing the slides by the air‐dry method. The yield of cells after CD19‐selection varied depending on the extent of disease and the amount of sample available for selection. Whenever possible, at least 200 interphase cells were scored for each probe. In samples with low cellular recovery, a minimum of 100 cells were required to be included in the study. The panel of probes (Abbott Molecular, Des Plaines, IL or Cytocell, Tarrytown, NY) for detecting specific chromosomal aberrations in B‐ALL, CLL/SLL, and other B‐cell neoplasms, and their respective normal variation cut‐offs are listed in Table 1.

**TABLE 1:** Summary of B‐cell neoplasms and FISH probes

Diagnosis^†^	Cases (PT/UD)^‡^	FISH Probes tested; chromosomal target	Normal variation^§^
B‐ALL	51 (51/0)	BCR/ABL dual fusion; t(9;22)(q34;q11.2)	1%
		D7S486/CEP7; 7q31/7p11.1‐q11.1	7q31: 2% 7p11.1‐q11.1: 1%
		TEL/AML1 (ETV6/RUNX1); 12p13.2/21q22	1%
		CEP4/CEP10/CEP17; (hyperdiploidy panel); 4p11‐q11/10p11‐q11/17p11.1‐q11.1	2%
		XY; Xp11‐q11/Yq12	1%
		TCF3/PBX1 dual fusion; 19p13.3/1q23	1%
		MLL break apart (KMT2A); 11q23	1%
		PRDM1/TNFAIP3/CEP6; 6q21/6q23.3/6p11.1‐q11	1%
		TP53/CEP17; 17p13.1/17p11.1‐q11.1	17p13.1: 4% 17p11.1‐q11.1: 1%
CLL/SLL	51 (38/13)	D13S319/13q34/CEP12; 13q14.3/13q34/12p11.1‐q11	13q14.3: 4% 13q34: 1% 12p11.1‐q11: 1%
		TP53/ATM; 17p13.1/11q22.3	17p13.1: 4% 11q22.3: 3%
		IGH break apart; 14q32	4%
		PRDM1/TNFAIP3/CEP6; 6q21/6q23.3/6p11.1‐q11	1%
MZL/SMZL	6 (2/4)	PRDM1/TNFIAP3/CEP6	See above
		D7S486/CEP7	See above
		D13S319/13q34/CEP12	See above
		TP53/ATM	See above
		IGH break apart	See above
		MALT1 break apart; 18q21	2%
		BCL6 break apart; 3q27	1%
MCL	9 (8/1)	IGH/CCND1 dual fusion; 14q32/11q13	1%
		D13S319/13q34/CEP12	See above
FL	5 (3/2)	IGH/BCL2; 14q32/18q21	1%
LPL	4 (1/3)	D13S319/13q34/CEP12	See above
B‐PLL	1 (0/1)	TP53/CEP17	See above
PTLD	1 (0/1)	IGH/MYC/CEP8; 14q32/8q24/8p11.1‐q11.1	1%

† B‐ALL, B‐lymphoblastic leukemia/lymphoma; CLL/SLL, chronic lymphocytic leukemia/small lymphocytic lymphoma; MZL/SMZL, marginal zone lymphoma/splenic marginal zone lymphoma; MCL, mantle cell lymphoma; FL, follicular lymphoma; LPL, lymphoplasmacytic lymphoma; PTLD, post‐transplant lymphoproliferative disorder; B‐PLL, B‐cell prolymphocytic leukemia.

‡ Number of cases including post‐therapy follow up/relapse (PT), and untreated disease (UD) samples.

§ Percentage range of normal variation (95% confidence interval) cut‐offs in our laboratory.

### 2.5 Molecular analysis

Immunoglobulin heavy chain (IGH) gene rearrangement (IGH PCR): Multiplexed fluorescent PCR amplification was performed on an ABI 3500xL genetic analyzer (Thermo Fisher Scientific, Waltham, MA), using six VH‐FR1 primers (tube A), seven VH‐FR2 primers (tube B), seven VH‐FR3 primers (tube C), six DH primers (tube D) and one DH7 primer (tube E), all with a consensus reverse JH primer (IdentiClone IGH Gene Clonality Assay, Invivoscribe Inc., San Diego, CA), followed by high‐resolution capillary electrophoresis and fragment analysis. The detection limit varied from 1 to 10% depending on the specific rearrangement.

BCR‐ABL1 RT‐PCR: Extracted RNA was subjected to quantitative reverse transcription real‐time PCR to simultaneously measure the quantity of the primary BCR‐ABL fusion transcripts b2a2, b3a2, and e1a2, using the Asuragen BCR/ABL1 Quant Assay (Asuragen, Austin, TX). Simultaneous PCR amplification of the ABL1 gene was performed as a control for sample RNA quality and as a reference for relative quantification.

### 2.6 Statistical analysis

Comparison of means and odds ratios were analyzed with MedCalc for Windows, version 19.2.0 (MedCalc Software, Ostend, Belgium).

## 3 RESULTS

### 3.1 Overall cohort characteristics

CD19S FISH was performed in a cohort of 128 samples from 88 patients over the study period. The male to female ratio was 3.7:1 and the median age was 65 years (range: 2–89 years). The samples were derived from bone marrow aspirates (85.2%) or peripheral blood (14.8%). The cohort largely comprised B‐ALL (39.8%, 51/128) and CLL/SLL (39.8%, 51/128) samples. Samples of a variety of other B‐cell neoplasms (20.3%, 26/128) accounted for the remainder (Table 1). Twenty‐five samples were obtained from untreated patients (Table 1), over half of which represented CLL/SLL (13/25). The remaining one hundred and three samples were obtained post‐therapy to determine disease status (including all B‐ALL samples).

### 3.2 Diagnostic versus post‐therapy sample characteristics

**FIGURE 1:**
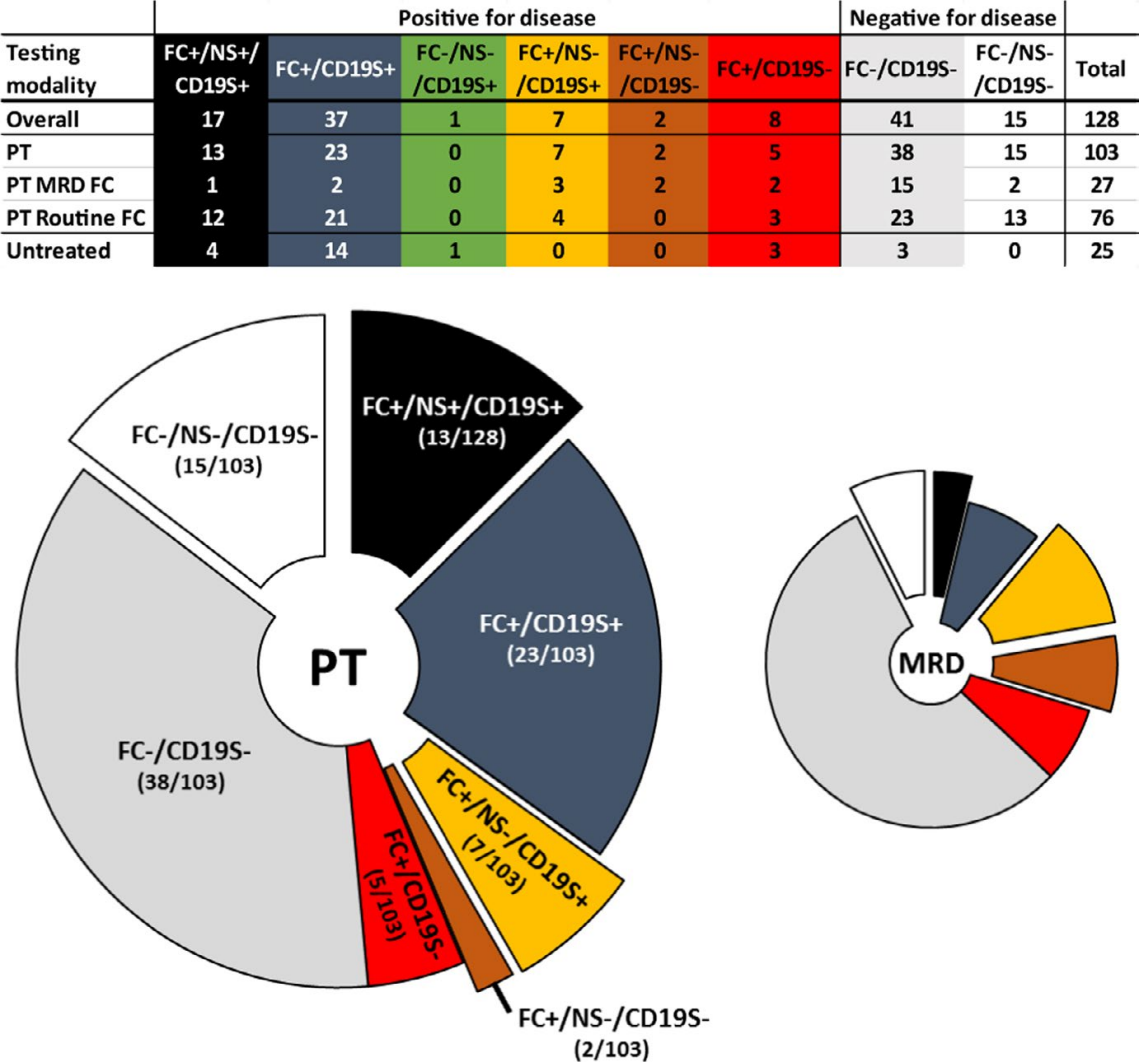
Relative proportion of cases assayed by the different testing modalities. Pie charts represent the relative proportion of cases assayed by FC (large chart includes both routine and MRD FC; small chart includes MRD FC only) and CD19S FISH, with or without NS FISH (“exploded” pie segments represent cohorts with NS FISH testing). Data are derived from the inset table and the corresponding ratios for segments in the large chart are given in parentheses. Positive (+) or negative (‐) detection by the type of testing modality is denoted. White and light gray segments represent the proportion of cases without detectable disease. Black and dark gray segments represent the proportion of positive cases detected by FC and CD19S FISH, with or without NS FISH testing, respectively. Gold segments represent the proportion of cases positive by CD19S FISH and negative by NS FISH. Red and brown segments represent the proportion of cases positive by FC but negative by CD19S FISH, with or without NS FISH testing, respectively

Routine FC detected disease in the majority of untreated (diagnostic) samples (84.0%, 21/25), while only 48.5% (50/103) of post‐therapy samples had disease detectable by FC (routine or MRD; Figure 1). CD19S FISH detected disease in 76.0% (19/25) of untreated samples, including one unique case where CD19S FISH detected disease (FL, 4.8% cells were positive for IGH/BCL2 translocation), while both NS FISH and routine FC did not (Figure 1, green column and Table S1, sample 1). IGH PCR analysis also detected a clonal IGH rearrangement in sample 1 (see below), further supporting the presence of disease in this sample (Table S1). Unfortunately, few untreated samples were tested by NS FISH (5/25 samples), precluding an accurate comparison of the ability of both FISH modalities to detect disease in untreated samples (Figure 1).

A variety of chemotherapeutic drug regimens were used to treat the different B‐cell neoplasms, which are listed in Table S1. Rituximab, a monoclonal antibody that targets CD20 antigen, was the most commonly used drug (44/103 samples; Table S1). Post‐Rituximab therapy samples that were positive for disease demonstrated no apparent decrease in the sensitivity of CD19S FISH (90.9%; 20/22 samples) compared to that in alternative therapy samples (82.1%; 23/28). The median duration elapsed after Rituximab treatment prior to positive disease sample collection was 6 months (range of 0.25 to 108 months elapsed), while the same statistic for negative disease samples was 7.25 months (range 0.25 to 125 months elapsed). Treatments directly targeting CD19 antigen (Blinatumomab or chimeric antigen receptor T‐cell therapy (CAR‐T)) were used in 12 cases (10 B‐ALL and two follicular lymphoma cases; Table S1). Specifically, 8 samples of B‐ALL were post single Blinatumomab therapy and serially derived from the same patient, of which 5 samples were positive for refractory disease by both FC (bright CD19 antigen) and CD19S FISH, including the first sample in the series (8 days post‐application). While the other 4 samples, comprising two samples of B‐ALL and two samples of FL, were post‐CAR‐T and negative for disease by both FC and CD19S FISH.

### 3.3 Concordance of molecular testing with FC and CD19S FISH

IGH PCR was performed in 21.9% (28/128) of the samples and BCR‐ABL1 RT‐PCR in four samples. BCR‐ABL1 RT‐PCR analyses were in complete agreement with FC and CD19S FISH results. IGH PCR analyses were overall in good agreement with results of FC and CD19S FISH, but in 14.3% (4/28) and 10.7% (3/28) of samples, IGH PCR results were discrepant from FC and CD19S FISH results, respectively. Notably, two molecular tests detected clonal peaks when FC was negative (Table S1; cells highlighted in gray): Sample 1 was concordant with CD19S FISH and was accordingly designated as a “positive” sample, whereas, sample 115 was discordant with both CD19S FISH and FC results and was designated a “negative” sample.

### 3.4 Comparison of CD19S FISH with NS FISH in post‐therapy samples

CD19S FISH was positive in 86.0% of all post‐therapy samples that had FC detectable disease (43/50; Figure 1), with the percent positive cells ranging from 2 to 99% (Table S1). NS FISH was performed in 44.0% of all positive samples (22/50) and detected disease in 59.1% (13/22; Figure 1), with percent positive cells ranging from 1.5 to 51.5% (Table S1). NS FISH did not detect disease in any samples negative by either FC or CD19S FISH.

**FIGURE 2:**
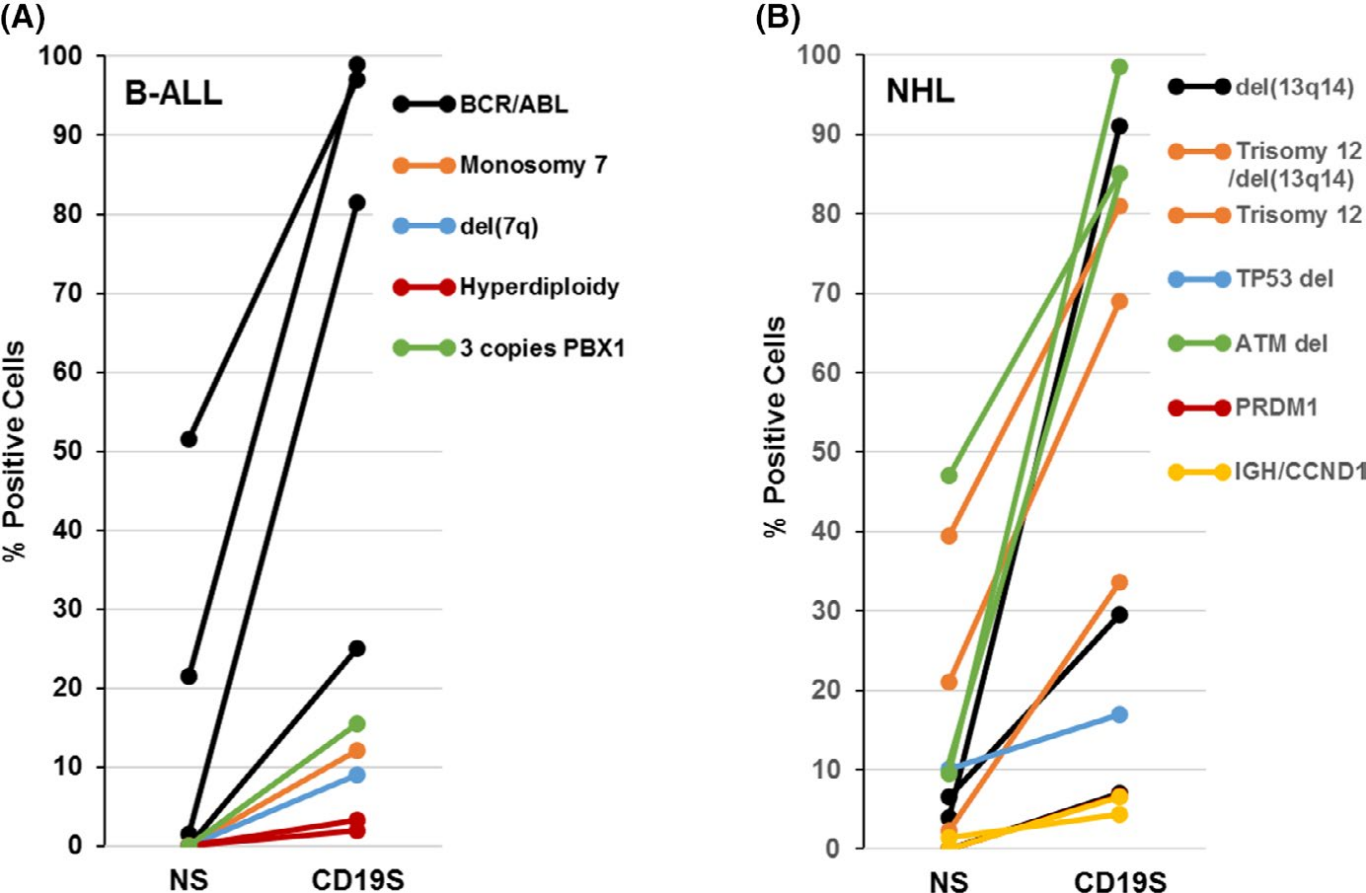
Comparison of FISH modalities. Percentage positive B‐ALL (A) and non‐Hodgkin lymphoma (B) cells as detected by NS and CD19S FISH. Specific chromosomal abnormalities are indicated by colored bars

Twenty‐two post‐therapy “positive” samples (comprising 25 chromosome aberrations) had data on both CD19S FISH and NS FISH, allowing direct comparison of these different FISH technique modalities in the detection of chromosome aberrations (Table 2). The average percentage of positive cells detected by NS FISH and by CD19S FISH was 10.10 ± 15.65% and 42.24 ± 38.23%, respectively (Table 2). CD19S FISH consistently detected a higher percentage of positive cells than NS FISH (Figure 2), and a comparison of means found a significantly increased rate of the detection of chromosomal aberrations by CD19S FISH compared to NS FISH (*p* < 0.001). Furthermore, within the subset of positive samples tested by both FISH modalities, 72.7% (8/11) of B‐ALL, 18.2% (2/11) of CLL/SLL, and 33.3% (1/3) of other B‐cell neoplasms had chromosomal aberrations that were uniquely detected by CD19S FISH (Table 2 and Figure 2). NS FISH did not effectively detect disease in B‐ALL samples with post‐therapy minimal disease (2/11 chromosome aberrations detected), but yielded informative results in other B‐cell neoplasms, particularly CLL/SLL (9/11 chromosome aberrations; Table 2).

**TABLE 2:** Summary of chromosomal aberrations in treated samples detected by all testing modalities (NS FISH, CD19S FISH, and FC) expressed as the percentage of cells

Diagnosis^†^	Chromosomal aberration	NS FISH	CD19S FISH	FC	FC mean ±SD
B‐ALL	BCR/ABL	0	25	0.1	0.30 ± 0.70
^‡^B‐ALL	Monosomy 7	0	12.1	0.2	
^‡^B‐ALL	del(7q)	0	9	0.2	
B‐ALL	Hyperdiploidy	0	2	^¶^0.08	
B‐ALL	Hyperdiploidy	0	0	^¶^0.039	
B‐ALL	Hyperdiploidy	0	0	^¶^0.02	
B‐ALL	Hyperdiploidy	0	3.3	^¶^0.016	
B‐ALL	3 copies PBX1	0	15.5	^¶^0.012	
CLL/SLL	del(13q14)	0	7	0.1	
CLL/SLL	PRDM1	0	6.7	0.1	
MCL	IGH/CCND1	0	6.5	2.4	
^‡^B‐ALL	BCR/ABL	1.6	81.5	0.2	18.15 ± 17.83
B‐ALL	BCR/ABL	51.5	97	30	
B‐ALL	BCR/ABL	21.5	99	^¶^14.749	
^§^CLL/SLL	del(13q14)	6.5	29.5	19	
CLL/SLL	del(13q14)	4	91	5	
^§^CLL/SLL	Trisomy 12 /del(13q14)	21	69	19	
CLL/SLL	Trisomy 12	39.5	81	4.9	
CLL/SLL	Trisomy 12	2.3	33.6	5	
CLL/SLL	TP53 del	10	17	65	
CLL/SLL	ATM del	9.5	98.5	21.5	
CLL/SLL	ATM del	47	85	38.5	
CLL/SLL	ATM del	11	84	25
MCL	IGH/CCND1	1.5	4.3	0.26
MZL	del(7q)	5.5	14	6
Mean ± SD		10.10 ± 15.65	42.24 ± 38.23		

† B‐ALL, B‐lymphoblastic leukemia/lymphoma; CLL/SLL, chronic lymphocytic leukemia/small lymphocytic lymphoma; MZL, marginal zone lymphoma; MCL, mantle cell lymphoma; FL, follicular lymphoma. Chromosomal aberrations detected by CD19S FISH and/or FC, but undetected by NS FISH are highlighted in gray.

‡ Chromosomal aberrations in the same B‐ALL sample.

§ Chromosomal aberrations in the same CLL/SLL sample.

¶ Disease detected by MRD FC.

### 3.5 Detection of rare subclones by CD19S FISH

After careful review of morphology (and in one case, after an additional selected FISH panel), no chromosomal aberration in this study was attributed to an underlying myeloid neoplastic clone. In the entire cohort (untreated and post‐therapy samples), 9 CLL/SLL, 5 B‐ALL (same patient), one SMZL, and one LPL sample had multiple recurrent chromosomal aberrations detectable by FISH, all other FISH positive samples had only one detectable aberration (Table S1). In these samples, CD19S FISH not only detected low‐level disease, but also detected persistent or emerging new subclonal abnormalities. For example, a B‐ALL sample with a BCR‐ABL t(9;22) stem‐line clone (detected by both NS and CD19S FISH), also contained rare (previously detected) subclones with monosomy 7 and deletion 7q, that in this instance were detected only by CD19S FISH (Table 2 and Table S1, Sample 5). Examples of emerging subclones in CLL/SLL samples uniquely detected only by CD19S FISH include: i) two subclones including one with ATM and TP53 deletions and one with MYC rearrangement, both likely derived from a stem‐line with an ATM deletion (Table S1, Sample 128), ii) a subclone with ATM deletion from a trisomy 12 stem‐line (Table S1, Sample 45), and iii) two subclones containing heterozygous deletion of 13q14, and 13q14 deletion plus trisomy 12, derived from a stem‐line with deletion TP53 (Table S1, Sample 77).

### 3.6 Comparison of FC and FISH in post‐therapy samples

Flow cytometry, either routine or B‐ALL MRD FC, was performed in the entire post‐therapy cohort and detected disease in 50 samples (Figure 1). Seven samples were positive for disease by FC (including four MRD flow samples), but negative by FISH (Figure 1). As stated earlier, NS FISH did not detect disease in any sample deemed negative by either FC or CD19S FISH. In post‐therapy samples that were positive by NS FISH, the average percentage of positive cells detected by FC was significantly higher compared to samples deemed negative by NS FISH (18.15 ± 17.83% vs. 0.30 ± 0.70%, *p* < 0.001), (Table 2). By extrapolation, this finding predicts that an FC positive cell detection of <1% will likely have a “negative” NS FISH outcome. Whereas, CD19S FISH detected disease in 81.8% of the negative NS samples (9/11; Table 2).

When MRD FC samples were excluded from the post‐therapy cohort, routine FC, CD19S FISH, and NS FISH sensitivities in detecting disease were 100 (40/40), 92.5 (37/40), and 75.0% (12/16), respectively. Thus, the odds ratio of CD19S FISH having a different outcome to routine FC was 0.132 (95% CI 0.007–2.647, *p* = 0.186), implying no significant difference in the sensitivity of these two methods. Whereas, the odds ratio of NS FISH having a different outcome to routine FC was 0.034 (95% CI 0.002–0.682, *p* = 0.027), signifying a statistically inferior sensitivity to routine FC in detecting disease.

### 3.7 Comparison of B‐ALL MRD FC and FISH in post‐therapy samples

A large proportion of B‐ALL samples was analyzed by MRD FC (26/51) and the majority were negative for disease (16/26, 61.5%; Figure 1). FISH analysis did not detect disease in any MRD FC negative sample. NS FISH was performed in 6 of the 10 positive samples, but detected disease in only one sample (sensitivity of 16.7%; Figure 1), whereas CD19S FISH detected disease in 6 of 10 samples (sensitivity of 60%; Figure 1). Although the number of positive MRD FC cases in our cohort is small (n = 10), CD19S FISH, although inferior to MRD‐FC, was again notably superior to NS FISH in detecting disease.

## 4 DISCUSSION

Prior studies have not systematically evaluated the diagnostic utility and applications of CD19S FISH in the detection of B‐cell neoplasms, particularly in comparison to NS FISH with concurrent standard FC studies. In this study, we demonstrate the advantages of CD19‐selection in detecting cytogenetic aberrations in post‐therapy samples of immature and mature B‐cell neoplasms. CD19S FISH significantly improved disease detection both quantitatively and qualitatively when compared to NS FISH and showed high sensitivity in disease detection statistically comparable to routine FC.

Our cohort was predominantly comprised of bone marrow aspirate samples obtained after therapy to determine disease status. Although the majority of samples were from B‐ALL and CLL/SLL patients, samples from patients with other types of B‐cell neoplasms were also represented. CD19S FISH proved to be advantageous over NS FISH in B‐ALL where most of the post‐therapy samples had a low disease burden (Figure 2). Conversely, many CLL/SLL samples were typically derived from untreated disease and contained numerous neoplastic cells, which were detectable by NS FISH in a high proportion of samples, making CD19S FISH selection less advantageous. However, within the subset of positive samples tested by both FISH modalities, a number of chromosomal aberrations and persistent/emerging rare subclones were uniquely detected only by CD19S FISH, while NS FISH did not detect disease in any CD19S FISH negative samples.

Analysis of the effect of direct CD19‐targeted therapies, which have the potential to down‐regulate CD19 surface antigen expression and thereby interfere with B‐cell selection and enrichment, was hindered by the small number of cases and largely inconclusive. The few post‐CAR‐T therapy samples were negative for disease by both CD19S FISH and FC, while Blinatumomab was applied once to a single patient and the post‐therapy samples had identical CD19S FISH and FC results. Interestingly, there are reports in the literature of concurrent down‐regulation or loss of CD19 surface antigen by monocyte‐mediated trogocytosis or “shaving” during Rituximab therapy.^18^ However, in our cohort, we found no evidence of decreased CD19S FISH sensitivity in the presence of this drug, even after the consideration of the duration of time elapsed from therapy application to sample collection.

On comparing the mean percent positive cells detected by standard FC of corresponding “negative” and positive NS FISH studies, statistically significant differences were noted. An FC detection level of <1% percent positive cells was highly predictive of a “negative” NS FISH result and such samples would potentially benefit from CD19S FISH. One caveat to this recommendation is that CD19S FISH does not reflect the actual disease burden of neoplastic B‐cells in a sample since these cells are sorted and enriched, and removed from the background cellular admixture. However, in conjunction with FC evaluation, CD19S FISH can provide useful information regarding the presence of residual or relapsed disease.

In general, interphase FISH evaluation of low‐level disease is limited by the probes available for disease detection and since all FISH probes have normal ranges of variation (ranging from 1 to 4% in our study) that can significantly affect the lower limit of disease detection. However, this is mitigated when enriching for the cells of interest. Accordingly, CD19S FISH demonstrated statistically comparable sensitivity to routine FC in our study, and in one instance CD19S FISH detected disease in an untreated sample (supported by molecular PCR analysis) when routine FC was negative. However, CD19S FISH had a notably inferior sensitivity to MRD FC for B‐ALL. This finding is in keeping with MRD FC as the current analytic gold standard for the detection of minimal measurable disease for B‐ALL^19^ and also plasma cell myeloma.^20^ Aside from the exquisite sensitivity (1/10,000 events in our laboratory), MRD FC concentrates cells of interest by the gating strategy, retaining background cells, and thereby can inform of the percentage of neoplastic cells in the whole sample. Other highly sensitive techniques that are commercially available for B cell MRD detection include next‐generation sequencing (NGS), which assesses clone‐associated IGH rearrangements and is considered to be an order of magnitude more sensitive than FC.^21^, ^22^ Drawbacks of NGS MRD include its considerably longer result turn‐around‐time, its relative expense, and its inability to detect evolving chromosomal aberrations. More investigative/experimental techniques such as “Immuno‐flowFISH,” which directly couples FC with FISH, may supersede existing techniques but are not currently available in daily hematopathology practice.^23^

While CD19S FISH has inferior sensitivity to MRD FC and is only a semi‐quantitative technique, it can complement FC in disease detection and contribute additional cytogenetic information important for risk stratification and disease management. Many examples of rare recurrent/persistent subclones or newly emerging subclones were detected in the present study by CD19S FISH alone. Subclones often arise under therapy selection pressure and invariably are associated with worse prognosis.^24^ The detection of rare emerging subclones reveals a qualitative advantage that CD19S FISH has over NS FISH and even standard FC. Moreover, CD19S samples are enriched with a relatively pure population of neoplastic B‐cells making them invaluable for additional molecular and genetic analyses.

Our study has some limitations. NS FISH was not performed in all cases, limiting the statistical power of comparisons between the two FISH modalities. Apart from CLL/SLL and B‐ALL samples, only a limited number of other types of B‐cell neoplasms were assessed, which precludes assessing the utility of CD19S FISH in such cases.

Despite these limitations, our study serves as proof‐of‐principle of the utility and advantages of interphase FISH analysis performed on CD19‐selected cells over non‐selected cells for the detection of recurrent cytogenetic aberrations in B‐cell neoplasms in the *post*‐*therapy* setting. In addition, the study shows that this FISH analysis modality appears unaffected by prior Rituximab treatment and complements and has sensitivity comparable to routine FC immunophenotypic analysis. Future studies with larger cohorts and a greater variety of B‐cell neoplasms are clearly warranted to further delineate the role of CD19S FISH in the diagnosis and follow‐up of B‐cell neoplasms.

## CONFLICTS OF INTEREST

No conflicts of interest.

## AUTHORS’ CONTRIBUTIONS

Conceptualization (AP & BA), Methodology/Investigation (VM, CW & AC), Formal analysis (AP), Writing (AP & BA), Review and Editing (AP, VM, GB, & BA), Visualization (AP), and Supervision (BA).

## Supporting information

Table S1

## Data Availability

Data generated or analyzed during this study are largely included in the article and its supplemental files. Any additional data that support the findings of this study are available upon request from the corresponding author. Additional data are not publicly available due to privacy or ethical restrictions.
