# Supplementary material for: Interphase fluorescence in situ hybridization analysis of CD19‐selected cells: Utility in detecting disease in post‐therapy samples of B‐cell neoplasms
Source: Cancer Med. 2021 Mar 15;10(8):2680–9. doi: 10.1002/cam4.3853 (PMC8026942; doi:10.1002/cam4.3853)
Supplement: Supplementary file 1 — Table S1 [file CAM4-10-2680-s001.docx]

| **Case** | **Age** | **Sex** | **^†^Therapy?** | **^‡^Specimen** | **Diagnosis** | **FISH Probe(s) used** | **^§^NS FISH (Disease % cellularity)** | **CD19S FISH (Disease % cellularity)** | **FC (Disease % cellularity)** | **^¶^Molecular** |  |
| --- | --- | --- | --- | --- | --- | --- | --- | --- | --- | --- | --- |
| 1 | 84 | M | Untreated | BM | FL | IGH/BCL2 | Negative | IGH/BCL2 (4.8%) | Negative | 322 FR1 |  |
| 2 | 77 | F | Ritux | BM | MZL | D7S486/CEP7 | 7q deletion (5.5%) | 7q deletion (14%) | 6 | 350 FR1, 288 FR2 |  |
| 3 | 57 | M | Benda/Ritux | PB | CLL | CLL panel | TP53 deletion (10%) | TP53 deletion (17%) | 65 | ND |  |
| 4 | 61 | M | SC/Ritux | BM | MCL | IGH/CCND1 | Negative | IGH/CCND1 (6.5%) | 2.4 | 388 FR1, 274 FR2 |  |
| 5 | 42 | F | SC | BM | B-ALL | BCR/ABL; D7S486/CEP7 | BCR/ABL (1.6%); D7S486/CEP7 (Negative) | BCR/ABL (81.5%), Monosomy 7 (12.1%), del(7q) (9%) | 0.2 | ND |  |
| 12 |  |  | SC/Blinat |  |  | BCR/ABL; XY (CD19S only) | Negative | BCR/ABL (25%), Trisomy X (28%) | <0.1 | ND |  |
| 16 |  |  |  |  |  | BCR/ABL; XY (CD19 only) | BCR/ABL (51.5%) | BCR/ABL (97%), Trisomy X (82.5%) | 30 | IS = 0.238 |  |
| 22 |  |  |  |  |  | BCR/ABL | Negative | Negative | Negative | ND |  |
| 47 |  |  |  |  |  | BCR/ABL; XY | ND | BCR/ABL (72%), Trisomy X (78%) | 0.37 MRD | ND |  |
| 49 |  |  |  |  |  | BCR/ABL; XY (CD19 only) | BCR/ABL (21.5%) | BCR/ABL (99%), Trisomy X (94%) | 14.749 MRD | ND |  |
| 66 |  |  |  |  |  | BCR/ABL | ND | Negative | Negative MRD | BCR/ABL negative |  |
| 90 |  |  |  |  |  | BCR/ABL | ND | Negative | Negative | ND |  |
| 100 |  |  |  |  |  | BCR/ABL | ND | BCR/ABL (96.5%) | 54 | ND |  |
| 6 | 70 | F | Lenal/Ibrut | BM | CLL | CLL Panel | ND | TP53 deletion (67.5%) | 15 | ND |  |
| 7 | 76 | M | Ponat | BM | B-ALL | BCR/ABL | Negative | Negative | Negative | ND |  |
| 8 | 14 | M | SC | BM | B-ALL | TEL/AML1 | Negative | Negative | Negative | ND |  |
| 9 | 58 | M | SC/Ibrut/Obinut/Ritux | BM | MCL | IGH/CCND1 | IGH/CCND1 (1.5%) | IGH/CCND1 (4.3%) | 0.26 | ND |  |
| 29 |  |  |  | BM | MCL | IGH/CCND1 | ND | Negative | 1 | Polyclonal |  |
| 10 | 65 | M | SC | BM | B-ALL | BCR/ABL | Negative | Negative | Negative | ND |  |
| 31 |  |  |  | BM | B-ALL | BCR/ABL | Negative | Negative | Negative | ND |  |
| 61 |  |  |  | PB | B-ALL | BCR/ABL | ND | Negative | Negative | ND |  |
| 84 |  |  |  | BM | B-ALL | BCR/ABL | ND | Negative | 0.003 MRD | Polyclonal |  |
| 105 |  |  |  | BM | B-ALL | BCR/ABL | ND | Negative | Negative MRD | ND |  |
| 123 |  |  |  | BM | B-ALL | BCR/ABL | ND | Negative | Negative MRD | ND |  |
| 11 | 4 | M | SC | BM | B-ALL | Hyperdiploidy panel | Negative | ALL Hyperdiploidy (3.3%) | 0.016 MRD | ND |  |
| 13 |  |  |  | BM | B-ALL | Hyperdiploidy panel | Negative | Negative | 0.039 MRD | ND |  |
| 34 |  |  |  | BM | B-ALL | Hyperdiploidy panel | Negative | Negative | 0.02 MRD | ND |  |
| 53 |  |  |  | BM | B-ALL | Hyperdiploidy panel | Negative | ALL Hyperploidy (2%) | 0.08 MRD | ND |  |
| 14 | 70 | M | Untreated | PB | LPL | D13S319/13q34/CEP12 | del(13q14) (7%) | del(13q14) (35.7%) | 14 | 329/344 FR1, 267/278 FR2, 142 FR3 |  |
| 79 |  |  | Untreated | BM | LPL | D13S319/13q34/CEP 12 | ND | Negative | 88.4 | 328/343 FR1, 267/278 FR2, 142 FR3 |  |
| 15 | 69 | M | Untreated | BM | CLL | D13S319/13q34/CEP12 | ND | Trisomy 12 (81%) | 54 | 343 FR1, 282 FR2, 143 FR3 |  |
| 17 | 80 | M | Ibrut | BM | CLL | D13S319/13q34/CEP12 | del(13q14) (6.5%), Trisomy 12/del(13q14) (21%) | del(13q14) (29.5%), Trisomy 12/del(13q14) (69%) | 19 | ND |  |
| 18 | 65 | M | Ibrut | BM | CLL | CLL panel | del(13q14) (4%) | del(13q14) (91%) | 5 | ND |  |
| 19 | 66 | M | Veneto/Ritux | BM | CLL | IGH | ND | Negative | Negative | ND |  |
| 20 | 73 | M | Benda/Ritux | BM | CLL | D13S319/13q34/CEP12 | ND | del13q14 (58.3%) | 16.5 | ND |  |
| 21 | 64 | M | Ibrut/Ritux | BM | CLL | D13S319/13q34/CEP12 | Negative | Negative | Negative | ND |  |
| 23 | 22 | F | SC/Ritux | BM | B-ALL | TCF3/PBX1; hyperdiploidy panel | ND | Negative | Negative MRD | ND |  |
| 24 | 79 | F | Untreated | BM | LPL | CLL panel; PRDM1/TNFAIP3/CEP6; IGH break apart | ND | del(6q) (94%), IGH rearrangement (8%) | 59.3 & 7.9 (subclone) | 355 FR1, 284 FR2, 143 FR3, 256 DH-JHlow |  |
| 25 | 68 | M | Benda/Ritux/Cirmt/Ibrut | BM | CLL | ATM/TP53 | ATM del (9.5%) | ATM del (98.5%) | 21.5 | ND |  |
| 26 | 60 | M | Untreated | BM | FL | IGH/BCL2 | ND | Negative | Negative | ND |  |
| 27 | 57 | M | Benda/Ritux | BM | CLL | PRDM1/TNFAIP3/CEP6 | Negative | PRDM1 del(6q21) (6.7%) | 0.1 | ND |  |
| 50 |  |  |  | BM | CLL | PRDM1/TNFAIP3/CEP6 | Negative | Negative | Negative | ND |  |
| 28 | 68 | M | Ritux | BM | CLL | D13S319/13q34/CEP12 | Trisomy 12 (39.5%) | Trisomy 12 (81%) | 4.9 | ND |  |
| 30 | 12 | M | SC | BM | B-ALL | BCR/ABL | Negative | Negative | Negative | ND |  |
| 48 |  |  |  | BM | B-ALL | BCR/ABL | ND | Negative | Negative MRD | ND |  |
| 87 |  |  |  | BM | B-ALL | BCR/ABL | ND | Negative | Negative MRD | ND |  |
| 121 |  |  |  | BM | B-ALL | BCR/ABL | ND | Negative | Negative MRD | Negative BCR/ABL |  |
| 32 | 74 | M | Untreated | PB | CLL | D13S319/13q34/CEP 12 | ND | Trisomy 12 (83.5%) | 13.3 | ND |  |
| 33 | 75 | M | Benda/Ritux/Obinut | BM | MCL | IGH/CCND1 | ND | Negative | Negative | Polyclonal |  |
| 35 | 49 | F | SC/Ritux | BM | MCL | IGH/CCND1 (NS & CD19S) and D13S319/13q34/CEP12 (NS) | Negative | Negative | Negative | Polyclonal |  |
| 103 |  |  |  | BM | MCL | IGH/CCND1 | ND | Negative | Negative | Polyclonal |  |
| 36 | 8 | F | Untreated | BM | PTLD | IGH/MYC/CEP8 | ND | Negative | Negative | ND |  |
| 37 | 9 | M | SC | BM | B-ALL | TCF3/PBX1 | Negative | 3x copies PBX1 (15.5%) | 0.012 MRD | ND |  |
| 40 |  |  |  | BM | B-ALL | TCF3/PBX1 | ND | 3x copies PBX1 (8%) | <0.4 | ND |  |
| 54 |  |  |  | BM | B-ALL | TCF3/PBX1 | ND | 3x copies PBX1 (5.5%) | 0.6 | ND |  |
| 81 |  |  |  | BM | B-ALL | TCF3/PBX1 | ND | Negative | Negative MRD | ND |  |
| 99 |  |  |  | BM | B-ALL | TCF3/PBX1 | ND | 3x copies PBX1 (98.5%) | 60 | ND |  |
| 106 |  |  |  | BM | B-ALL | TCF3/PBX1 | ND | 3x copies PBX1 (17%) | 20.3 MRD | ND |  |
| 38 | 70 | M | Untreated | PB | CLL | CLL panel | del(13q14) (11%) | del(13q14) (22%) | 40 | ND |  |
| 39 | 52 | M | SC/Ritux | BM | CLL | D13S319/13q34/CEP 12 | Trisomy 12 (2.3%) | Trisomy 12 (33.6%) | 5 | ND |  |
| 41 | 72 | M | Untreated | PB | CLL | D13S319/13q34 | ND | del(13q) (89%) | 67 | ND |  |
| 42 | 56 | F | Untreated | BM | CLL | D13S319/13q34 | ND | del(13q) (99%) | 90 | 334 FR1, 273 FR2, 133 FR3, 209 DH-JH |  |
| 116 |  |  | Ibrut/Ritux | PB | CLL | ALK break apart, TP53/ATM | ND | 3x copies ALK (77.5%); ATM deletion (3.5%) | 33.8 | ND |  |
| 43 | 70 | F | Ibrut | BM | MZL | MALT1 break apart | Negative | Negative | Negative | ND |  |
| 44 | 12 | F | CAR-T | BM | B-ALL | Hyperdiploidy panel | Negative | Negative | Negative | ND |  |
| 45 | 70 | M | Untreated | BM | CLL | CLL panel | ND | Trisomy 12 (72.8%), ATM del (5%) | 82.5 | ND |  |
| 78 |  |  | Benda/Ritux | BM | CLL | D13S319/13q34/CEP 12 | ND | del(13q14) (54.5%) | 6 | 341 FR1, 275 FR2, 139 FR3, 255 DH-JHlow |  |
| 95 |  |  | Benda/Veneto/Ritux | BM | CLL | D13S319/13q34/CEP12 | ND | Negative | Negative | ND |  |
| 119 |  |  |  | BM | CLL | D13S319/CEP12 | ND | Negative | Negative | ND |  |
| 46 | 61 | M | Benda/Ritux/Obinut | BM | CLL | D13S319/13q34/CEP 12 (CD19S only); TP53/ATM (NS only) | ATM del 47% | del13q14 (85%) | 38.5 | ND |  |
| 51 | 51 | M | CAR-T | BM | FL | IGH/BCL2 | Negative | Negative | Negative | Polyclonal |  |
| 52 | 32 | M | SC/Ritux | BM | B-ALL | BCR/ABL | Negative | Negative | Negative | Negative BCR/ABL |  |
| 55 | 64 | F | Untreated | PB | MZL | BCL6 break apart | 3x copies BCL6 (5.5%) | 3x copies BCL6 (92.5%) | 6.8 | ND |  |
| 56 | 81 | M | Untreated | BM | MCL | IGH/CCND1 | ND | Negative | Negative | ND |  |
| 57 | 61 | M | Benda/Ritux/Veneto | BM | CLL | D13S319/13q34/CEP12 | ND | del(13q14) (10.5%) | 0.24 | ND |  |
| 109 |  |  |  | BM | CLL | D13S319/13q34 | ND | Negative | Negative | Polyclonal |  |
| 58 | 52 | F | Untreated | BM | CLL | D13S319/13q34/CEP12 | ND | del(13q14), (94.5%) | 95 | ND |  |
| 59 | 8 | F | SC | BM | B-ALL | Hyperdiploidy panel | ND | Negative | Negative | Polyclonal |  |
| 76 |  |  |  | BM | B-ALL | Hyperdiploidy panel | ND | Negative | Negative MRD | ND |  |
| 60 | 78 | M | Veneto/Ritux | BM | CLL | D13S319/13q34/CEP12 | Negative | del(13q14) (homozygous 7.0%) | <0.1 | 331/352 FR1, 242/257 FR2, 11/119 FR3, 109 DH-Jhlow |  |
| 62 | 50 | M | SC/Ritux | BM | B-ALL | MLL break apart | Negative | Negative | Negative MRD | ND |  |
| 69 |  |  |  | BM | B-ALL | MLL break apart | ND | Negative | Negative MRD | ND |  |
| 125 |  |  |  | BM | B-ALL | MLL break apart | ND | Negative | Negative MRD | ND |  |
| 63 | 62 | M | Untreated | PB | MZL | BCL6 break apart | ND | Negative | 7 | 323 FR1, 258 FR2, 115 FR3, 270 DH-JH |  |
| 64 | 54 | M | Benda/Ritux/Obinut/Cerdul/CAR-T | BM | FL | IGH/BCL2 | ND | Negative | Negative | Polyclonal |  |
| 65 | 3 | F | SC | BM | B-ALL | TEL/AML1 | ND | Negative | <0.5 | ND |  |
| 68 |  |  |  | BM | B-ALL | TEL/AML1 | ND | Negative | Negative MRD | ND |  |
| 67 | 5 | M | SC | BM | B-ALL | ALL Hyperdiploidy (Adult) | ND | Negative | Negative MRD | ND |  |
| 73 |  |  |  | BM | B-ALL | Hyperdiploidy panel | ND | Negative | Negative MRD | ND |  |
| 70 | 63 | M | Ibrut | BM | CLL | D13S319/13q34/CEP12 | ND | Trisomy 12 (55%), del(13q14) (30.5%) | 4.3 | ND |  |
| 71 | 75 | M | Untreated | BM | CLL | D13S319/13q34/CEP12 | ND | Trisomy 12 (93%) | 58 | ND |  |
| 72 | 62 | F | Untreated | BM | CLL | CLL panel | ND | del(13q14) (97%), 5'IGH del (88%) | 78.6 | ND |  |
| 74 | 2 | F | SC | BM | B-ALL | Hyperdiploidy panel | ND | Negative | Negative | ND |  |
| 75 | 76 | M | Obinut | BM | CLL | CLL panel | ND | 13q14 homozygous deletion | 1.5 | ND |  |
| 77 | 60 | M | Ibrut | BM | CLL | CLL panel | ND | del(13q14) (heterozygous) (29%), del(13q14) (heterozygous) & trisomy 12 (4.5%), and del(13q14) (homozygous) & trisomy 12 (3.5%); TP53 deletion (99.5%) | 40 | 330 FR1, 260 FR2, 119 FR3, 171 DH-Jhlow |  |
| 80 | 62 | M | Untreated | BM | B-PLL | TP53/CEP 17 | ND | TP53 deletion (98.5%) | 85.5 | 332 FR1, 267 FR2, 131 FR3, 137 DH-JHlow, 383DH-Jhhigh |  |
| 82 | 73 | M | SC/CAR-T | BM | B-ALL | BCR/ABL | ND | Negative | Negative | ND |  |
| 83 | 4 | F | SC | BM | B-ALL | Hyperdiploidy panel | ND | Negative | Negative MRD | ND |  |
| 85 | 88 | M | Ritux | BM | CLL | D13S319/13q34/CEP12 | ND | Negative | Negative | ND |  |
| 88 | 71 | M | Benda/Ritux/Ibrut | PB | CLL | D13S319/13q34/CEP 12, BCL3 break apart | ND | Trisomy 12 (88%); BCL3 (91%) | 80 | ND |  |
| 89 | 83 | M | Acala | PB | CLL | CLL panel | ND | del(13q14) (98.5%) | 97 | ND |  |
| 91 | 71 | F | Fludara/Ritux | PB | CLL | D13S319/13q34/CEP12; IGH break apart; TCL1 break apart | ND | Negative | 30 | ND |  |
| 92 | 80 | M | SC/Ritux | PB | FL | IGH/BCL2; ATM/TP53 | ND | Negative | Negative | ND |  |
| 93 | 60 | M | Untreated | BM | CLL | D13S319/13q34/CEP12 | ND | del(13q14) (85%) | 88 | ND |  |
| 94 | 12 | M | SC | BM | B-ALL | CDKN2A/CEP 9 | ND | Negative | Negative | ND |  |
| 96 | 61 | M | Ibrut/Ritux | BM | CLL | D13S319/13q34/CEP12 | ND | Negative | Negative | ND |  |
| 97 | 3 | M | SC | BM | B-ALL | TEL/AML1 | ND | Negative | Negative | ND |  |
| 118 |  |  |  | BM | B-ALL | TEL/AML1 | ND | Negative | Negative | ND |  |
| 98 | 76 | M | Untreated | BM | CLL | D13S319/13q34/CEP12 | ND | Trisomy 12 (80.5%) | 41 | 328/338 FR1, 263/273 FR2, 120/138 FR3, 216 DH-Jhlow |  |
| 101 | 76 | M | SC/Ritux/Ibrut | BM | CLL | TP53/ATM | ND | TP53 deletion (97%) | 73 | ND |  |
| 102 | 70 | M | Ritux/Benda/Bortez/Lenol | BM | MCL | IGH/CCND1 | ND | IGH/CCND1 (7%) | 0.65 | ND |  |
| 108 |  |  |  | BM | MCL | IGH/CCND1 | ND | Negative | Negative | ND |  |
| 104 | 75 | M | Ibrut | BM | CLL | PRDM1/TNFAIP3/CEP6 | ND | PRDM1 deletion (30%) | 22 | ND |  |
| 107 | 68 | F | SC/Benda/Ritux | BM | LPL | TP53/CEP17 | ND | Negative | Negative | ND |  |
| 110 | 72 | M | Idela/Veneto | BM | CLL | D13S319/CEP12 | ND | Trisomy 12 (1.3%) | 0.06 | ND |  |
| 111 | 66 | M | Benda/Ritux | PB | CLL | D13S319/13q34 | ND | del(13q14) (75.5%) | 1.5 | ND |  |
| 112 | 67 | F | Untreated | PB | CLL | D13S319/CEP12, ATM/TP53 | ND | Negative | 8 | ND |  |
| 113 | 75 | F | SC/Ritux | BM | CLL | PRDM1/TNFAIP3/CEP6 | ND | PRDM1 deletion (6.5%) | 92 | 352 FR1, 292 FR2, 152 FR3 |  |
| 114 | 60 | M | Untreated | PB | SMZL | MALT1, BCL6 break apart, D7S486/CEP 7 | ND | 7q31 deletion (77%), 3x copies BCL6 (77.5%), BCL6 rearrangement (7.5%) | 59.2 | 355 FR1, 294 FR2, 155 FR3, 235 DH-Jhlow |  |
| 115 | 74 | M | Benda/Ritux/Veneto | BM | CLL | D13S319/CEP12 | ND | Negative | Negative | 344 FR1, 272 FR2, 131 FR3 |  |
| 117 | 79 | F | Veneto/Ibrut | BM | CLL | D13S319/CEP12 | ND | Negative | Negative | ND |  |
| 120 | 80 | F | Untreated | PB | MZL | D13S319/CEP 12, TP53/ATM, BCL6 break apart, MALT1 break apart, CCND1 break apart | ND | Trisomy 12 (25.5%) | 3 | 347 FR1, 275 FR2, 125 FR3 |  |
| 122 | 61 | F | Ibrut | BM | CLL | D13S319/CEP 12, TP53/ATM | ND | Deletion 13q14 (92.5%) | 78 | ND |  |
| 124 | 78 | F | Untreated | PB | CLL | CLL panel | Gain of 13q34 (5.5%), gain of 13q34 & 13q14 deletion (7.0%) | Gain of 13q34 (43.5%), gain of 13q34 & 13q14 deletion (46%) | 15 | ND |  |
| 126 | 8 | F | SC | BM | B-ALL | TEL/AML1 | Negative | Negative | Negative MRD | ND |  |
| 127 | 89 | M | SC/Ritux | PB | CLL | D13S319/CEP12 | ND | Negative | Negative | Polyclonal |  |
| 128 | 79 | M | Benda/Ritux/Cirmt/Ibrut | BM | CLL | D13S319/CEP 12 (NS only), TP53/ATM, MYC break apart (CD19S only) | ATM deletion (11%), 13q14 deletion (2.7%) | ATM deletion (84%), ATM & TP53 deletions (3%), MYC rearrangement (13%) | 25 | ND |  |
| 129 | 5 | F | SC | BM | B-ALL | ETV6 (TEL)/(RUNX1 (AML1) | ND | Negative | 0.003 MRD | ND |  |
| ^†^Ritux = Rituxumab, Benda = Bendamustine, SC = Standard chemotherapy, Blinat = Blinatumomab, Lenal = Lenalidomide, Ibrut = Ibrutinib, Ponat = Ponatinib, Obinut = Obinutuzomab, Veneto = Venetoclax, Cirmt = Cirmtuzumab, CAR-T = chimeric antigen receptor T-cell therapy, Cerdul = Cerdulatinib, Acala = Acalabrutinib, Fludara = Fludarabine, Bortez = Bortezomib, Idela = Idelalisib | | | | | | | | | | | |
| ^‡^BM = Bone marrow, PB = peripheral blood | | | | | | | | | | | |
| ^§^ND = Not determined | | | | | | | | | | | |
| ^¶^IS = International Scale for BCR-ABL | | | | | | | | | | | |
